# Study of the Effect of Treatment With Atrial Natriuretic Peptide (ANP) and Cinaciguat in Chronic Hypoxic Neonatal Lambs on Residual Strain and Microstructure of the Arteries

**DOI:** 10.3389/fbioe.2020.590488

**Published:** 2020-11-10

**Authors:** Alvaro Navarrete, Zhuoming Chen, Pedro Aranda, Daniel Poblete, Andrés Utrera, Claudio M. García-Herrera, Alejandro Gonzalez-Candia, Felipe A. Beñaldo, German Ebensperger, Roberto V. Reyes, Emilio A. Herrera, Anibal J. Llanos

**Affiliations:** ^1^Departamento de Ingeniería Mecánica, Universidad de Santiago de Chile, Santiago, Chile; ^2^Instituto de Ciencias de la Salud, Universidad de O’Higgins, Rancagua, Chile; ^3^Pathophysiology Program, Faculty of Medicine, Institute of Biomedical Sciences (ICBM), Universidad de Chile, Santiago, Chile; ^4^International Center for Andean Studies (INCAS), Universidad de Chile, Santiago, Chile

**Keywords:** chronic hypoxia, ring opening test, pre-stretching test, residual deformation, histology, pulmonary hypertension

## Abstract

In this study, we assessed the effects of Atrial Natriuretic Peptide (ANP) and Cinaciguat, as experimental medicines to treat neonatal lambs exposed to chronic hypoxic conditions. To compare the different treatments, the mechanical responses of aorta, carotid, and femoral arterial walls were analyzed by means of axial pre-stretch and ring-opening tests, through a study with *n* = 6 animals for each group analyzed. The axial pre-stretch test measures the level of shortening in different zones of the arteries when extracted from lambs, while the ring-opening test is used to quantify the degree of residual circumferential deformation in a given zone of an artery. In addition, histological studies were carried out to measure elastin, collagen, and smooth muscle cell (SMC) nuclei densities, both in control and treated groups. The results show that mechanical response is related with histological results, specifically in the proximal abdominal aorta (PAA) and distal carotid zones (DCA), where the cell nuclei content is related to a decrease of residual deformations. The opening angle and the elastic fibers of the aorta artery were statistically correlated (*p* < 0.05). Specifically, in PAA zone, there are significant differences of opening angle and cell nuclei density values between control and treated groups (*p*-values to opening angle: Control-ANP = 2 ⋅ 10^–2^, Control-Cinaciguat = 1 ⋅ 10^–2^; *p*-values to cell nuclei density: Control-ANP = 5 ⋅ 10^–4^, Control-Cinaciguat = 2 ⋅ 10^–2^). Respect to distal carotid zone (DCA), significant differences between Control and Cinaciguat groups were observed to opening angle (*p*-value = 4 ⋅ 10^–2^), and cell nuclei density (*p*-value = 1 ⋅ 10^–2^). Our findings add evidence that medical treatments may have effects on the mechanical responses of arterial walls and should be taken into account when evaluating the complete medical outcome.

## Introduction

High altitude hypoxia is characterized by a decrease in environmental O_2_ partial pressure (PaO_2_), due to a drop of barometric pressure (Abdalla and Will, 1995; Julian, 2011). This effect is physiologically and clinically relevant over 2,500 m above sea level (m.a.s.l), due to a drop in arterial oxygen pressure (PaO_2_) and saturation (SaO_2_) (Julian, 2011). It is estimated that more than 170 million people live at more than 2,500 m.a.s.l around the world. ∼80 million of them in Asia and ∼40 million in South America (Andean mountains), where the highest population density is found above 3,500 m.a.s.l. (Penaloza, 2012; Herrera et al., 2015). The biological response to this condition leads to a reduction of inspired and alveolar oxygen pressure, which results in a decreased oxygen concentration in the blood (arterial hypoxemia) (Virués-Ortega et al., 2006). Long-term exposure induces physiological compensations such as alveolar hyperventilation and erythropoiesis that promotes oxygen transport; however, these changes are insufficient to achieve the same conditions as at sea level (Julian, 2011). Maladaptive responses could have as consequence many cardiovascular diseases, such as acute mountain sickness, pulmonary edema, subacute mountain sickness in children, or sleep apnea, all of them related to some degree of high blood pressure or cardiovascular impairment (Penaloza, 2012).

Chronic hypoxia is one of the factors that cause pulmonary hypertension, which affects to a greater degree the lungs of fetuses and newborns compared to adults, and significant effects on cardiovascular function can be seen (Ke et al., 2017). In particular, gestation under high-altitude conditions promotes postnatal pulmonary hypertension, due to cardiopulmonary dysfunction and remodeling with persistent effects even at sea level (Herrera et al., 2010) and risk of heart malformation (Penaloza and Arias-Stella, 2007; Steinhorn, 2010; Fuloria and Aschner, 2017). At birth, as a result of exposure to chronic hypoxia, the small-resistant pulmonary arteries do not dilate adequately, favoring a structural luminal narrowing and a corresponding increase of vascular tone, resulting in hypoxemia and pulmonary hypertension (Gao and Raj, 2011; Rol et al., 2017). This phenomenon, known as persistent pulmonary hypertension of the newborn (PPHN), is a failure of normal circulatory transition at birth, where pulmonary vascular resistance (PVR) remains abnormally elevated (Chetan et al., 2007; Sharma et al., 2015). In lowland populations, less than 1% of the neonates have respiratory distress syndrome, while in highland population (>2,500 m.a.s.l.), this condition can reach up to 10%, including PPHN (Astorga et al., 2018). Furthermore, high-altitude induced other responses such as an increased heart rate and contraction force, enhancing cardiac output (Palmer et al., 1999; Keyes et al., 2003; Chen et al., 2006; Huicho and Niermeyer, 2006; Penaloza and Arias-Stella, 2007; Herrera et al., 2010; Castillo-Galán et al., 2016). This increases oxygen demand and consumption, and induces biomechanical alterations in the cardiopulmonary system, such as heart hypertrophy and/or thickening of the arterial walls (Rouwet et al., 2002; Skilton et al., 2005). This phenomenon leads to a decrease of elastic fibers and an increase of collagen, decreasing the elastic capacity and hindering blood circulation and oxygen transmission to the organs and extremities (Rouwet et al., 2002; Thompson et al., 2011). Herrera et al. (2011) reveal that the fetuses of rats gestated in hypoxic chambers with 13% O_2_ (normal condition 21% O_2_), equivalent to being between 3,700 and 4,000 m.a.s.l., present hypertrophy remodeling of the aorta, and that early fetal hypoxia reduces the contractile response of the artery to potassium (K^+^), in contrast with other studies that reveal that late fetal hypoxia increases the contractile sensitivity to potassium (K^+^).

Until 1990, there was no drug-based treatment approved for pulmonary hypertension, but in the last 20 years medicines like Nifedipine, Diltiazem, Veletri, sodium Warfarin, or Bosentan have been produced, which help reduce the symptoms and prevents cardiovascular accidents, mainly by decreasing the development of blood clots and increasing pulmonary vasodilatation. However, these treatments are useful in only some cases, and most of them require medical follow-up because the secondary effects can produce other complications, such as spontaneous hemorrhage or poor liver functioning (The Cleveland Clinic, 2020). Therefore, there is still a need to search for new treatments. In this study, we evaluated the effects of the experimental drugs ANP and Cinaciguat, as pulmonary vasodilators, that activate soluble guanylate cyclase (GCs) (Pandey, 2005; Chester et al., 2009, 2011; Salinas et al., 2010). Atrial natriuretic peptide (ANP) activates guanylate cyclase A (GC-A), a natriuretic peptide receptor A(NPR-A), to increase the intracellular concentration of cyclic guanosine monophosphate (cGMP), resulting in multiple favorable cardiovascular effects, such as vasodilation (promotes smooth muscle cell relaxation, including dilation of veins and arteries), diuresis, inhibition of cell growth and sympathetic activity, and lowering of venous return. The pharmacokinetics of ANP are characterized by a short plasma half-life and high clearance. Two phases are observed in the plasma disappearance curve (two compartments) in a range of dose (Tan et al., 1993). Cinaciguat (BAY 58–2667) is a class of soluble guanylate cyclase activator molecule. The NO-sGC pathway is disrupted in the conditions of oxidative stress, such as during pulmonary hypertension of the neonate by chronic hypoxia. Oxidation of the heme group on sGC renders the enzyme insensitive to endogenous NO and exogenous nitro-vasodilators. Cinaciguat preferentially activates the heme-oxidized, NO-resistant form of sGC. As a result, Cinaciguat induces vasodilation, increasing intracellular cGMP levels.

To quantify the behavior of pathological tissue, several biomechanical *ex vivo* assessments, such as uniaxial tensile, wire myograph, pressurization (inflation–extension), and biaxial tests have been established (Karimi et al., 2013; Pichamuthu et al., 2013; Cañas et al., 2017). Su et al. (2018) studied the impact of chronic hypoxia on pulmonary artery according to its mechanical properties and wave propagation capacity. In particular, uniaxial tensile test to rupture and under loading-unloading cycles were performed to determine arterial stiffness and viscoelastic properties on normoxic and hypoxic groups; from this analysis, significant differences were observed in viscoelasticity behavior, which decreased in hypoxic groups. Golob et al. (2017) perform uniaxial tensile test in axial and circumferential direction in pulmonary arteries to evidence strain- and remodeling-induced stiffening by chronic pulmonary hypertension, where significant difference in high-strain stiffness modulus parameter was observed between normoxic and hypertensive groups. Wang et al. (2013) by means of static and sinusoidal pressure-inflation test performed in pulmonary artery, in order to determine differences in viscoelastic behavior of chronic hypoxia-induced pulmonary hypertension and normoxic condition, concluding that static and some dynamic parameters experiment significant changes under pulmonary hypertension condition. By other side, numerical modeling is a technique widely used to describe mechanical response of arterial tissue under different conditions, where implemented models require experimental information obtained by means of the biomechanical test mentioned below (Labrosse et al., 2009; García-Herrera et al., 2012, 2016; Cañas et al., 2018). In this sense (de Gelidi et al., 2017), has determined the effect of thickness and critical levels of pressure on aneurysm formation, using uniaxial tensile test and a finite element analysis of the pressurization-inflation test.

One way of quantifying changes in the biomechanical response of the material is by residual deformation tests, which provides information on the passive stretching state in which the blood vessels are found (Liu and Fung, 1988). To determine residual longitudinal deformations, an axial pre-stretch test has been carried out (Vonavková and Horný, 2020), which measures the contraction undergone by the vessels once removed from the body. Brossollet and Vito, 1995 showed that the axial *ex vivo* shortening level is independent of the state of muscular activation, because it postulates that the muscle cells are oriented circumferentially. There are many biomechanical changes in the arteries produced by aging or by diseases that are manifested as a marked decrease of the pre-stretch and are generally attributed to elastic fibers impairment (Horný et al., 2017). In order to quantify stress level in circumferential direction in arteries, opening angle test has been carried as a well-known procedure in biological structures (Destrade et al., 2017). García-Herrera et al. (2016) studied the circumferential residual stress distribution thorough the ring-opening test in human ascending aortas, both in healthy groups and in groups with Marfan syndrome, ascending aortic aneurysm, and bicuspid valve, where residual stress exhibits statistical differences of healthy groups with respect to pathological groups. Focusing in studies of chronic hypoxia-induced pulmonary hypertension using ring opening test procedure (Tian et al., 2011), linked opening angle, geometry and histological results in pulmonary artery to establish how arterial remodeling influences in hypertensive groups, determining a decrement in opening angle respect to normoxic group in rats. Li et al. (2004) by means a number of parameters, including opening angle between them, determine main parameters in recovery of pulmonary artery remodeling, when blood pressure is lowered; hypothesizing that blood pressure is linearly related with structural parameters along with opening angle through empirical formulas. Moreover, the ring-opening angle in arteries can be affected by changes in the levels of muscle activation or by microstructural changes in the arterial wall (Rachev et al., 2000). It has been shown that the level of residual deformation decreases in the arteries of animals with prolonged hypertension, and this is directly related to increased stiffness and decrease of elastin (Tian et al., 2011). Fung and Liu (1991) showed that hypoxia affects the residual circumferential deformations, altering the vascular function of the main arteries. Although many studies have been made on residual deformations, there is very little information relating the effect of hypoxia and hypertension on the behavior of residual arterial deformations in mammals, and in particular there is no information regarding axial pre-stretch with the hypoxic condition and with treatments to prevent this medical condition.

According to the Cinaciguat and ANP effects, we proposed as null hypothesis that there is no biomechanical or structural differences between the studied groups. Our alternative hypothesis is that the treatment will decreased arterial thickness and stiffness in the treated animals. Therefore, the objective of this research is to quantify the effect of a neonatal treatment with the vasodilator drugs Cinaciguat (Chester et al., 2009) and ANP (Sehgel et al., 2015) on the arterial geometry, residual longitudinal and circumferential deformations, supported by histological analyses of the aorta, carotid, and femoral arteries of sheep gestated and born at 3,600 m.a.s.l. with chronic hypoxia and pulmonary hypertension. A detailed analysis of the axial and ring opening tests were attempted here, segmenting the arteries into various parts to assess the deformation field across the arteries.

The biological material studied, the experimental procedures, geometrical measurement, and the different experiments developed are described in section “Materials and Methods.” In section “Results” the measurements of the initial geometry for different arteries is made, and experimental results of mechanical tests and histological studies are shown. In section “Discussion,” the relationship between the results obtained are discussed, to determine the effectiveness of the different treatments applied.

## Materials and Methods

The Faculty of Medicine Ethics Committee of the Universidad de Chile approved all experimental procedures (Protocol CBA#694 FMUCH). The studies on animals were performed according to the Guide for the Care and Use of Laboratory Animals published by the United States National Institutes of Health (NIH Publication No. 85-23, revised 1996) and adheres to the American Physiological Society’s Guiding Principles in the Care and Use of Animals.

### Animals

The studied arteries were obtained from fifteen newborn sheep (*Ovis aries*) aged 15 days old, all of them gestated, born and raised at high altitude (INCAS Research Station, Putre, 3,600 m) with chronic hypoxia and pulmonary hypertension. Since birth, every animal was measured and weighed daily, until euthanasia. Three groups were set: (1) Control group, treated with vehicle (NaCl 0.9%, 1 ml/kg), (2) Cinaciguat group (35 μg/kg in 1 ml/kg), and (3) Atrial natriuretic peptide group (ANP) (5 μg/kg in 1 ml/kg). All treatments were given daily during 7 days after the seventh day of life. Information of body weight at the beginning (day 7 of life) and at the end of the treatment (day 14 of life) is provided in Table 1, showing similar weight between the different groups. From each animal, the aorta, carotid, and femoral arteries were used for the mechanical tests.

**TABLE 1:** Body weight at the beginning (day 7 of life) and at the end of the treatment (day 14 of life).

Body weight (Kg)			

Age (*d*)	Control	ANP	Cinaciguat
7	4.36 ± 0.18	4.53 ± 0.19	4.30 ± 0.20
14	5.67 ± 0.36	5.90 ± 0.25	5.68 ± 0.29

In relation to sheep model, it has been used to study the normal and abnormal cardiovascular development of the fetus and the neonate for almost a century, and more recently to study the programming of cardiopulmonary function. In this sense, the sheep neonate shows similar hemodynamic responses to developmental hypoxia, as those seen in humans. Although is a more expensive and difficult model to raise, it is worth due to various reasons. When compared to other animal models such as rodents, the neonatal vascular development and structural characteristics of babies (human neonates) share much more characteristics with sheep. These characteristics are well reviewed elsewhere (Morrison et al., 2018), and includes similar developmental stages, size of the neonate, organs and tissues, and *in vivo* heart rate and arterial pressure, among others. Therefore, the neonatal lamb model represents similarities observed in cardiovascular development and function with humans, and it is a well-recognized translational model (Gonzaléz-Candia et al., 2020).

### Experimental Procedure

#### Tested Arterial Tissue

**FIGURE 1:**
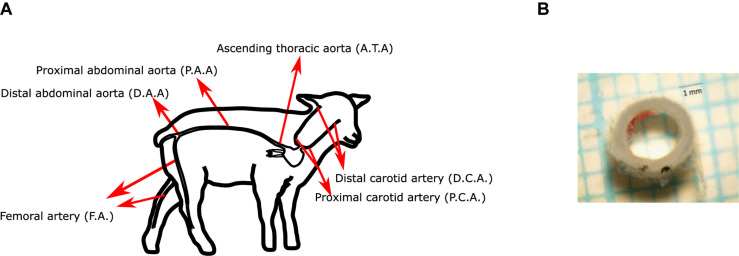
**(A)** Scheme of the arteries studied in this work. **(B)** Representative image of an arterial segment.

In this research, aorta, carotid and femoral arteries have been studied. The segments were defined and named as follows (Figure 1A):

– **Ascending thoracic aorta (ATA):** First segment of the thoracic aorta, from heart to diaphragm.
– **Proximal abdominal aorta (PAA):** Segment from diaphragm to right renal artery.
– **Distal abdominal aorta (DAA):** Segment from right renal artery to external iliac artery.
– **Femoral artery (FA):** Arterial segment from the inguinal canal to the saphenous artery (above the patella).
– **Carotid artery:** the common carotid artery was determined starting from the brachicephalic artery to the base of the inferior maxilar bone. The artery was divided in 2 segments of equal length: proximal (PCA; closer to the heart) and distal (DCA; closer to the head).

All mechanical tests were performed in a post mortem time interval of 1 h approximately, after the euthanasia procedure. Once the arteries have been extracted from the animal bodies, axial pre-stretching test was performed, and then rings were cut and kept in cold saline. Then, with the *ToupView software*, these rings were photographed, obtaining the thickness and diameter of the arteries (Figure 1B).

#### Axial Pre-stretch Test

**FIGURE 2:**
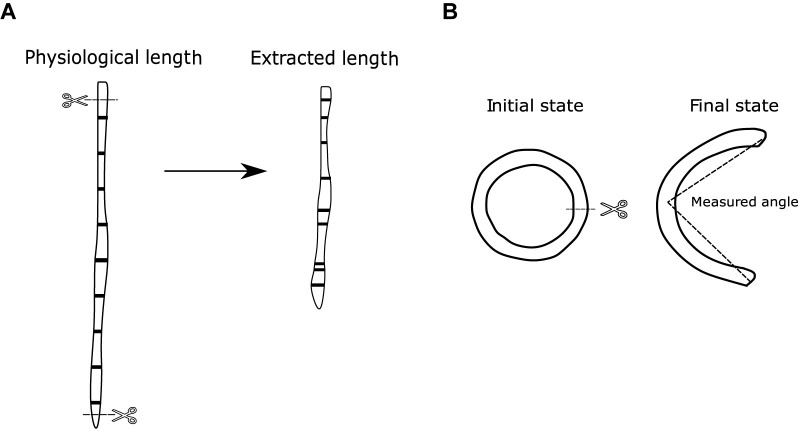
**(A)** Axial pre-stretch test scheme. **(B)** Ring opening test scheme.

To observe the residual longitudinal deformations of the arteries under *in vivo* conditions, it was necessary to perform the axial pre-stretch test, which consists in measuring the length of the artery *in situ*, and compare it with the length after extraction, without the intervention of the adjacent organs. The arteries were marked *in situ* at given distances with black gel pencil 0.7 mm thick ball-point and photographed. To observe the elastic retraction undergone along each marked sector, a photograph was taken 15 min after been extracted while keeping the marked arteries in saline (Figure 2A).

The abdominal aorta was thoroughly cleaned and the distance between two marks, *in situ* and after the excision, was determined. Marks were made just below the renal arteries and above the aortoiliac bifurcation. The aortas were divided in five segments of approximately 50 mm each, the carotids in seven parts of approximately 20 mm each, and the femoral in six segments of approximately 10 mm each. Subsequently, the arteries were thoroughly removed and the distances between each mark was measured. Due to length variations between animals, the segmentations were not always equal between subjects of the same group. This was solved using an interpolation of the measured pre-stretch with respect to equal percentage segments that are defined relative to the full length of each artery.

#### Ring Opening Test

For the observation of the residual circumferential deformations of the aorta, carotid, and femoral arteries, the same rings extracted to measure thickness and diameter of the arteries are used in this assay. 2 mm long rings were extracted for each zone (Cañas et al., 2018), which are indicated in Figure 1A. Three of the samples were meant for testing and one for histology. No samples from the distal section were used due to the large number of branches in it.

The cut rings were immersed in normal saline solution at 39°C for 10 min, and then they were cut radially and kept in the same solution for 20 min, so the residual deformations would become stabilized. Once the rings had opened, they were photographed, and their opening angle was determined. The measurement of the opening angle was made by joining the ends of the cut at an approximation of what would be the middle point of the already open artery (Figure 2B). Further details of the methodology can be found in García-Herrera et al. (2016).

#### Histology

**FIGURE 3:**
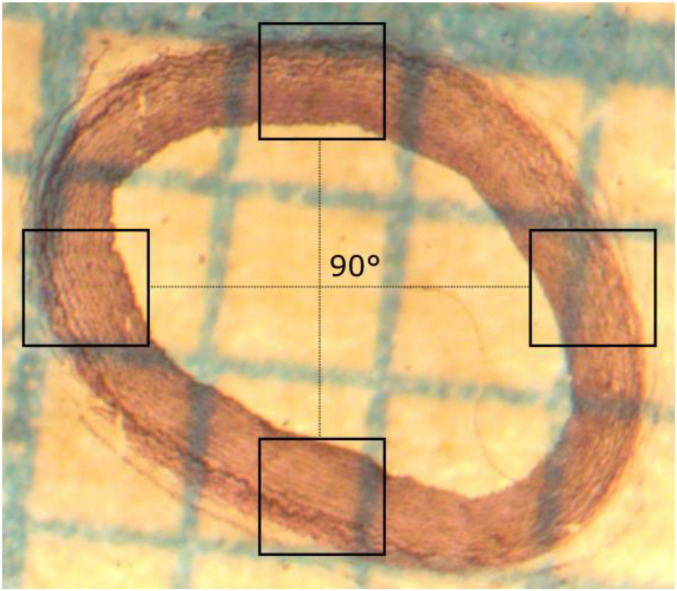
Scheme of four characteristic zones where micrographs were collected, each at a 90° angle to the other.

Changes in mechanical properties of arteries are related to variation in the collagen, elastin, or smooth muscle cell (SMC) densities. The quantification of each component provides a better understanding of changes in mechanical properties, which can be indication of potential vascular disease (Kochová et al., 2012). In this sense, histological procedure is performed to determine if there is any change in the arterial wall structure in the different experimental groups. The histological procedure was carried out using ring segments of aorta, carotid, and femoral arteries. The first step of this procedure consists in tissue fixation with 4% formaldehyde for 24 h to preserve their composition, avoiding damage during any histological process and keeping its structure intact. Then, the samples were washed in PBS 1x, embedded in paraffin and cut in 5 μm thickness serial sections and placed on slides (Astorga et al., 2018). Finally, these slides were stained with the Van Gieson and elastic Van Gieson staining procedures, in order to identify the amount of collagen, elastin and cell nuclei density from photographs of the analyzed samples. For each arterial zone, 2–3 samples were generated and analyzed to perform histological analysis. From each sample, four zones were photographed, separated by 90° from each other. The measurements in each zone were repeated 5 times and from these measurements we get an average per each animal (Figure 3).

**FIGURE 4:**
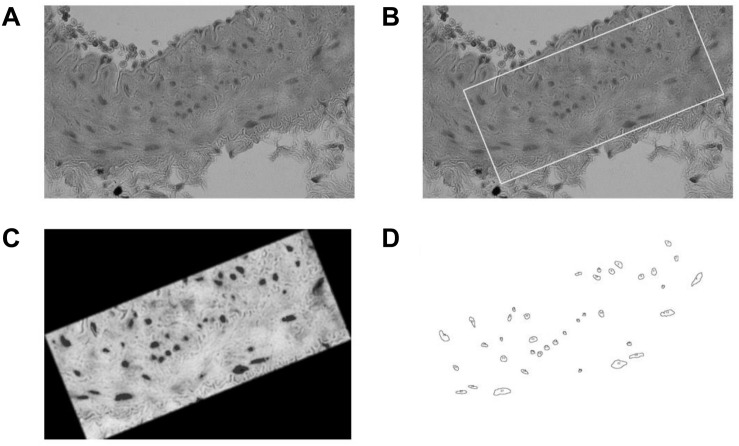
Counting protocol: **(A)** Histology image sample. **(B)** ROI selection within media layer. **(C)** Color thresholding. **(D)** Particle analyzer result.

The cell nuclei profile counting strategy was carried out using ImageJ software. The first stage of this method is the selection of a region of interest (ROI) within limits of tunica media, and then apply gaussian blur and grayscale filters. Once the nuclei are easily distinguishable, a thresholding filter is applied to select the visible nuclei. Then, a built-in binary-watershed method is applied to isolate the cells that are connected to adjacent ones. After a visual confirmation of the previous procedure, and the correspondent adjustments if necessary, the Particle Analyzer plugin is used to quantify the cell contours, identifying the total number of cells within ROI. This procedure is described in Figure 4. The main disadvantage of this approach is the fact that 2D densities of 3D objects are been measured, so the calculated values could be biased in relation to a more accurate methods, like stereology studies (Tschanz et al., 2014).

A similar method was applied for the elastic fiber quantification, which was made using the Color Deconvolution plugin of ImageJ software. The first step is the selection of the elastin and collagen main representative colors in small ROI areas, to obtain a stain-matrix which is valid for the color and illumination profiles of each image sample. Once the stain matrix was identified, the Color Deconvolution plugin is applied in a ROI within the media layer of the sample, obtaining for each image, the main elastin and collagen color maps, with a third residual color image. From there, a thresholding method is used to measure the main color pixel selection, allowing to estimate the elastin and collagen contents by making a pixel division between the threshold pixel selection and the ROI total pixel area.

Histological images were also used to measure the thickness of each arterial layer, where the percentage of tunica media layer thickness relative to total thickness was determined.

### Statistical Analysis

Values are expressed as mean ± SEM. Six animals were studied for each group analyzed (Control, ANP, and Cinaciguat treatment). All the analyses were carried out with Graphpad Prism 6.01 (GraphPad Software Inc., San Diego, CA, United States) software, and comparisons where were performed by Student’s *t*-test. Statistical significance was considered when *p* < 0.05 (Glagov et al., 1987), Meanwhile correlation analysis was performed via Pearson’s correlation test (*R*^2^), where null hypothesis indicates that the data corresponds to a population in which there is no correlation between the two variables analyzed.

## Results

### Geometric Data

Table 2 shows the average measurements of the geometry of the tested arteries in all groups: arterial thickness and equivalent internal diameter determined from internal perimeter of the arteries, assuming circumferential geometry of the artery section.

**TABLE 2:** Thickness and internal diameter of the specimens for all groups and arteries.

	Control		ANP		Cinaciguat	
Arterial Segment	Thickness (mm)	Internal diameter (mm)	Thickness (mm)	Internal diameter (mm)	Thickness (mm)	Internal diameter (mm)
ATA	1.93 ± 0.27	6.09 ± 0.75	2.16 ± 0.22	6.01 ± 0.64	2.35 ± 0.41	6.18 ± 0.73
PAA	1.07 ± 0.19	4.93 ± 0.56	1.06 ± 0.06	5.16 ± 0.36	1.03 ± 0.12	5.24 ± 0.50
DAA	0.86 ± 0.07	4.19 ± 0.34	0.94 ± 0.17	4.05 ± 0.61	0.86 ± 0.07	4.64 ± 0.52
PCA	0.52 ± 0.08	2.03 ± 0.33	0.58 ± 0.11	2.14 ± 0.22	0.52 ± 0.08	2.03 ± 0.33
DCA	0.70 ± 0.06	2.07 ± 0.33	0.73 ± 0.16	2.10 ± 0.24	0.70 ± 0.06	2.07 ± 0.23
FA	0.41 ± 0.58	1.87 ± 0.26	0.52 ± 0.79	1.72 ± 0.62	0.41 ± 0.06	1.87 ± 0.26

*Values are expressed as mean ± SEM. ATA, Ascending thoracic aorta; PAA, Proximal abdominal aorta; DAA, Distal abdominal aorta; PCA, Proximal carotid artery; DCA, Distal carotid artery; FA, Femoral artery. There are no significative differences between groups according statistical analysis.*

### Axial Pre-stretch

#### Aorta

**FIGURE 5:**
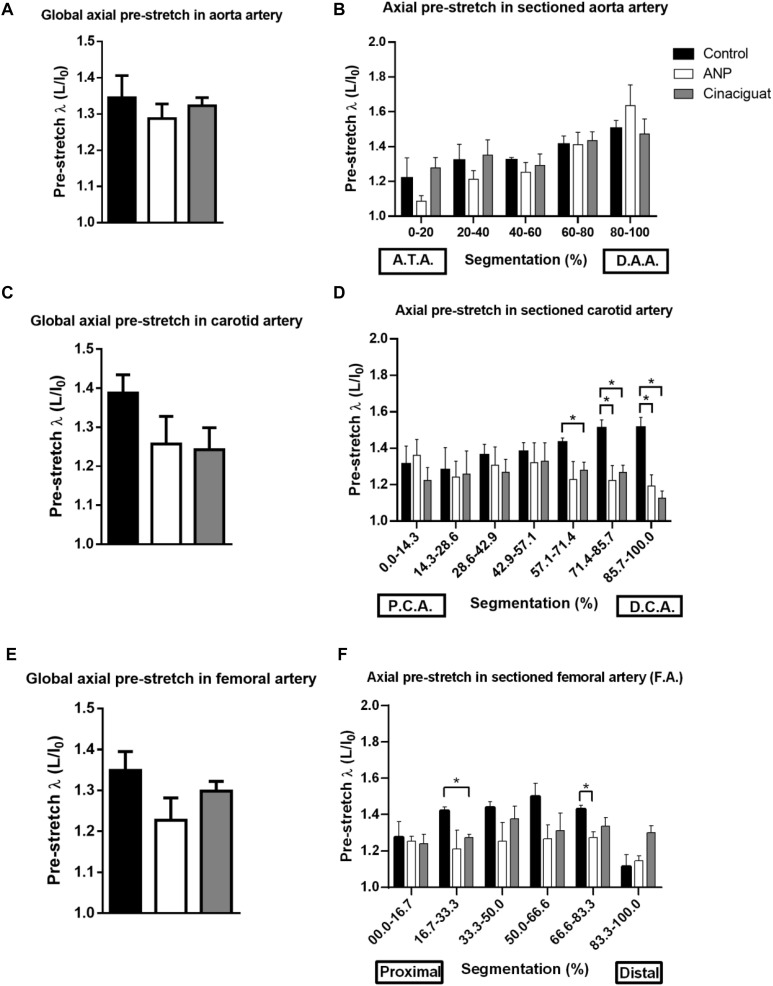
Axial pre-stretch results. **(A)** Global axial pre-stretch of the aorta (number of samples: Control = 3, ANP = 5, Cinaciguat = 4). **(B)** Average axial pre-stretch of the aorta, segmented 5 times from the ascending thoracic aorta to the distal abdominal aorta. **(C)** Global axial pre-stretch of carotid artery (number of samples: Control = 3, ANP = 5, Cinaciguat = 4). **(D)** Average axial pre-stretch of the carotid, segmented 7 times from the proximal (PCA) to the distal zone (DCA). **(E)** Global axial pre-stretch of the femoral artery (number of samples: Control = 2, ANP = 4, Cinaciguat = 4). **(F)** Average axial pre-stretch of the femoral arteries, segmented 6 times from the proximal to the distal zone. Groups are Control (black), ANP (white) and Cinaciguat (gray). Values are expressed as mean ± SEM. Significant differences (*p* ≤ 0.05, *t*-student) between groups are represented in square brackets.

Figure 5A shows the average measurements of global axial pre-stretch of the aorta, and Figure 5B the axial pre-lengthening according to each segment in which the artery was divided (see section “Axial Pre-stretch Test”), from ascending thoracic aorta (ATA) to distal abdominal aorta section (DAA). The bars correspond to average ± SEM values of each group.

According to Figure 5A, the level of global average pre-stretching is around 1.3 in aorta artery, without differences between control and treated groups, according to the *t*-student analysis. However, global results show heterogeneous behavior of pre-stretching along aorta arteries that were measured, going from 1.09 ± 0.03 in ANP group as minimum pre-stretching value in proximal zone, to 1.64 ± 0.12 in ANP group as maximum pre-stretching value in distal zone. Figure 5B evidences that the axial pre-stretch of the aorta shows non-homogeneous behavior over its entire length and this value is continuously increasing from the proximal to the distal zone of the artery. Specifically, in control group, pre-stretch value in ascending thoracic zone (ATA) is 1.218 ± 0.018, while in distal abdominal zone, this value corresponds to 1.504 ± 0.047, which reflects an average increment of 23.48%. For ANP and Cinaciguat treatments, same behavior is observed, where percentage increase of pre-stretching between proximal and distal zone of the aorta is 50.64 and 15.26%, respectively. Significant differences for ANP group were observed between ascending thoracic zone (0–20%) and abdominal sections of aorta artery (zones denoted by 40–60%, 60–80%, and 80–100%, according to Figure 5B). No differences were observed between control and treated groups.

#### Carotid Artery

Figure 5C shows the overall average axial pre-stretch of the carotid arteries, and Figure 5D shows the average axial pre-stretch going from distal (DCA) to proximal sector (PCA).

Global pre-stretching in carotid arteries (Figure 5C) states upper average values to control group, with a value of 1.39 ± 0.05, in comparison with ANP and Cinaciguat group (1.26 ± 0.07 and 1.24 ± 0.03, respectively), but there are not significant differences between them. A better visualization to explain the reason of this level of pre-stretching in control group is given by Figure 5D, where a considerable difference of pre-stretching values is observed comparing treated groups relative to controls in distal carotid zone (DCA). In fact, in fifth and seventh segment there is significant statically differences, where pre-stretching value in the sixth segment of the control group is 1.51 ± 0.05, while reached values in ANP and Cinaciguat groups are 1.22 ± 0.08 and 1.27 ± 0.04, respectively. In the seventh segment, pre-stretching in control group is 1.51 ± 0.06, while ANP and Cinaciguat groups reveals significant differences respect to non-treated group (1.19 ± 0.06 and 1.13 ± 0.04, respectively).

#### Femoral Artery

Global pre-stretch in femoral arteries is shown in Figure 5E, while pre-stretching in segmented and Figure 5F the axial pre-lengthening according to each segment.

Global pre-stretching values (Figure 5E) does not show significant differences between treated and control groups, with average values ranging from 1.2 to 1.35 approximately. In local pre-stretching values (Figure 5F), there are isolated significant differences between control and Cinaciguat groups in sector denoted by segmentation 16.7–33.3% (1.42 ± 0.02 and 1.27 ± 0.02, respectively), along with control and ANP group in sector of 66.6–83.3% segmentation (1.43 ± 0.20 and 1.27 ± 0.03, respectively).

### Ring Opening

#### Aorta

**FIGURE 6:**
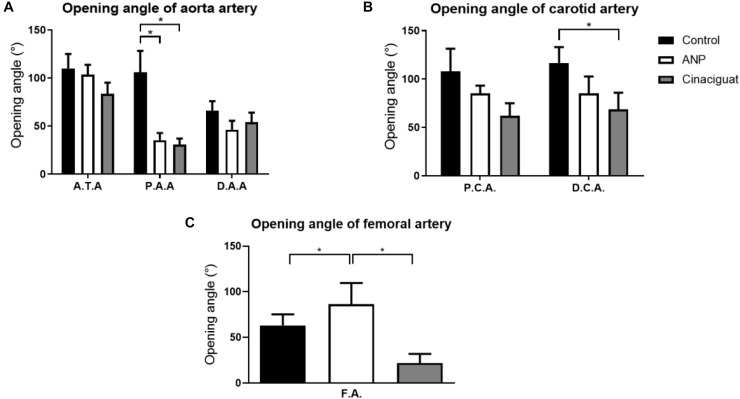
Ring-opening test results. **(A)** Opening angle of aorta, according to its group and sector, where ATA, Ascending thoracic Aorta (number of samples: Control = 6, ANP = 6, Cinaciguat = 6); PAA, Proximal abdominal Aorta (number of samples: Control = 6, ANP = 5, Cinaciguat = 6); DAA, Distal abdominal Aorta (number of samples: Control = 6, ANP = 6, Cinaciguat = 6). **(B)** Opening angle of the carotid arteries, according to their group and sector (number of samples in PCA and DCA: Control = 6, ANP = 6, Cinaciguat = 6). **(C)** Opening angle in femoral arteries, with their corresponding groups (number of samples FA: Control = 4, ANP = 6, Cinaciguat = 6). Values are expressed as mean ± SEM. Significant differences (*p* ≤ 0.05, *t*-student) between groups are represented in square brackets.

The results obtained from the ring opening of the aorta are shown in Figure 6A. In all samples, there is a maximum opening angle in ascending thoracic zone (ATA) and it decreases going to PAA or DAA according to group studied. As example of this fact, in control group, opening angle in ATA section is 110.06 ± 14.983°, in PAA is 106.00 ± 22.339° and to DAA is 66.00 ± 9.911°. In ATA and DAA zones, significant differences between control and the treated groups are not observed, except in the PAA sector, where the opening angle drops from 106.00 ± 22.34° to 35.33 ± 7.48° and 30.79 ± 6.25°, for the ANP and Cinaciguat groups, respectively.

#### Carotid Artery

The results obtained for the ring opening of the carotids are shown in Figure 6B. In general, there are not statistical changes in the opening angles for the different carotid zones; however, average opening angle values experiment a decrease in treated groups respect to control group. Significant differences are observed in distal sector of Cinaciguat group, where the mean opening angle drops 41.08% with respect to the control group value.

#### Femoral Artery

Finally, the results of the femoral arteries are shown in Figure 6C. Femoral arteries (FA) undergo different behaviors under ANP and Cinaciguat groups relative to the control group. While the ANP group shows increased opening angle (86.49 ± 23.12°) with respect to the control group (63.24 ± 12.07°), without significant differences, the Cinaciguat group exhibited a statistically significant decrease compared to the non-treated group (21.92 ± 10.02°).

### Histological Analysis

**FIGURE 7:**
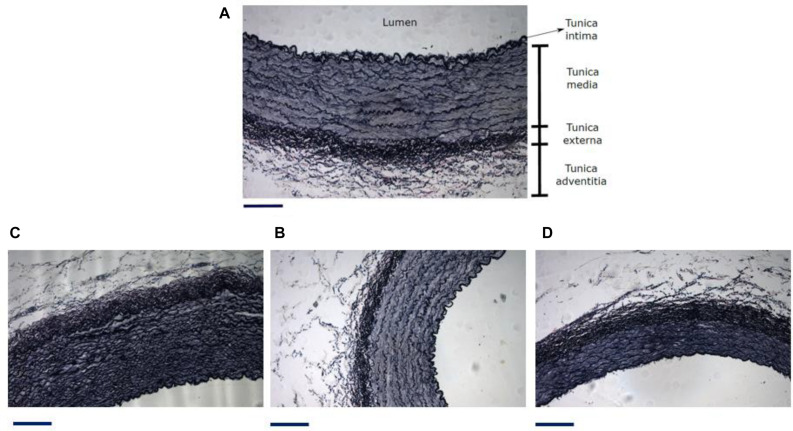
**(A)** Different zones and layers distinguishable in an artery. Representative micrograph of the Elastic Van Gieson **(B)** proximal abdominal aorta (PAA), **(C)** proximal carotid artery (PCA) and **(D)** femoral artery (FA). Black line under each image is a scale bar indicating a reference of 200 μm. From histological results it is possible to get information about the geometrical measurements of each artery layer and the arterial composition. Table 3 shows tunica media thickness (denoted as media) and the sum of tunica externa and tunica adventitia thickness (denoted as adventitia) (Witter et al., 2017), ratio between area luminal and vascular, and percentage content of elastin and collagen in cross section of aorta, carotid and femoral arteries. The content of elastic fibers was made inside the media layer.

According to histological procedure, detailed in section “Histology”, main results are obtained from this technique in Table 3. Figure 7A, describes characteristic layers observable in arterial wall, representative micrographs of different analyzed zones are seen in Figures 7B–D.

**TABLE 3:** Media (tunica media) and adventitia (tunica externa and tunica adventitia; Witter et al., 2017) thickness, and percentage ratio between area luminal and vascular: **(A)** aorta artery; **(B)** carotid and femoral arteries. Percentage of collagen fibers and elastin in the arteries’ cross section: **(C)** aorta artery; **(D)** carotid and femoral arteries.

(A)									

Arterial segment	ATA (ascending thoracic aorta)			PAA (proximal abdominal aorta)			DAA (distal abdominal aorta)		
Group	Control	ANP	Cinaciguat	Control	ANP	Cinaciguat	Control	ANP	Cinaciguat
Media thickness (mm)	0.96 ± 0.15	0.93 ± 0.12	0.83 ± 0.16	0.53 ± 0.05(*)	0.49 ± 0.01	0.39 ± 0.03 (*)	0.39 ± 0.03	0.40 ± 0.05	0.46 ± 0.04
Adventitia thickness (mm)	0.50 ± 0.06	0.59 ± 0.03	0.57 ± 0.07	0.40 ± 0.05	0.42 ± 0.07	0.36 ± 0.02	0.36 ± 0.03	0.39 ± 0.08	0.41 ± 0.02
% Area luminal/vascular	48.9 ± 3.1	43.9 ± 2.3	46.1 ± 7.2	48.5 ± 1.6(*)	51.9 ± 2.0	58.9 ± 1.0 (*)	52.6 ± 3.1	49.9 ± 5.3	54.8 ± 1.6

**(B)**									

**Arterial segment**	**Distal carotid artery (DCA)**			**Proximal carotid artery (PCA)**			**Femoral (FA)**		
**Group**	**Control**	**ANP**	**Cinaciguat**	**Control**	**ANP**	**Cinaciguat**	**Control**	**ANP**	**Cinaciguat**

Media thickness (mm)	0.31 ± 0.03	0.40 ± 0.03	0.27 ± 0.02	0.23 ± 0.01	0.27 ± 0.03	0.25 ± 0.03	0.33 ± 0.04	0.33 ± 0.07	0.20 ± 0.05
Adventitia thickness (mm)	0.26 ± 0.02	0.32 ± 0.02	0.26 ± 0.04	0.27 ± 0.02	0.24 ± 0.02	0.25 ± 0.00	0.22 ± 0.05	0.23 ± 0.02	0.20 ± 0.04
% Area luminal/vascular	41.5 ± 1.3	35.3 ± 2.5	42.5 ± 1.5	47.4 ± 1.2	44.6 ± 3.3	43.1 ± 3.3	44.6 ± 8.4	40.7 ± 9.0	47.1 ± 3.6

**(C)**									

**Arterial segment**	**ATA (ascending thoracic aorta)**			**PAA (proximal abdominal aorta)**			**DAA (distal abdominal aorta)**		
**Group**	**Control**	**ANP**	**Cinaciguat**	**Control**	**ANP**	**Cinaciguat**	**Control**	**ANP**	**Cinaciguat**

% Elastin	19.13 ± 2.64(*)	26.27 ± 1.87	29.28 ± 1.31(*)	34.15 ± 6.66	39.44 ± 1.90	37.62 ± 4.28	41.19 ± 2.81	36.66 ± 2.22	38.12 ± 2.04
% Collagen	3.15 ± 0.66	2.94 ± 1.02	4.13 ± 0.51	8.03 ± 0.41(*)	3.06 ± 0.40(*, +)	8.07 ± 1.42(+)	9.07 ± 0.36(*)	7.26 ± 0.56(*)	9.71 ± 1.00

**(D)**									

**Arterial segment**	**Distal carotid artery (DCA)**			**Proximal carotid artery (PCA)**			**Femoral (FA)**		
**Group**	**Control**	**ANP**	**Cinaciguat**	**Control**	**ANP**	**Cinaciguat**	**Control**	**ANP**	**Cinaciguat**

% Elastin	26.21 ± 1.89	25.73 ± 3.06	23.79 ± 5.13	31.43 ± 1.67	29.04 ± 2.69	33.62 ± 1.41	31.42 ± 2.20	31.39 ± 2.65	26.60 ± 2.84
% Collagen	12.30 ± 1.13	8.91 ± 1.36	10.65 ± 1.11	7.06 ± 0.95	6.65 ± 1.96	8.17 ± 0.91	11.70 ± 1.81(*)	7.16 ± 1.14	4.94 ± 1.52(*)

ATA, Ascending thoracic aorta; PAA, Proximal abdominal aorta; DAA, Distal abdominal aorta; FA, Femoral artery; PCA, Proximal carotid artery; DCA, Distal carotid artery. Values are expressed as mean ± SEM. Significant differences (p ≤ 0.05, t-student): a matching pair of symbols (* and +) indicate the groups that present differences.

**FIGURE 8:**
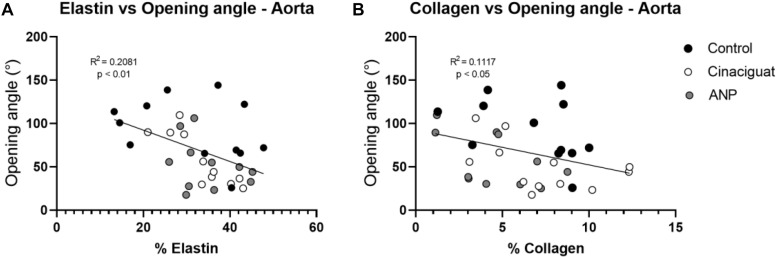
Correlation analysis between biomechanical and histological results. **(A)** elastin vs. opening angle. **(B)** Collagen vs. opening angle. Groups are Control (black), ANP (white) and Cinaciguat (gray).

In general, there are no significant differences between treated and untreated groups, exception of for elastin percentage in ATA zone, between the control and Cinaciguat groups, and collagen percentage between the control and ANP groups in the PAA and DAA sectors. Differences in the collagen concentration were found in the PAA zone between the treated groups (Table 3C). In Table 3D a significant difference is seen in collagen content between the control and Cinaciguat groups in the femoral zone (FA). Correlation analysis between fiber densities and opening angle (Figure 8) shows the relation between these two variables in the aorta artery, mixing all the studied zones of this artery.

Tables 4A,B show the average counts of muscle cell nuclei of the aorta, carotid, and femoral arteries for each sector and animal studied in the ring opening assays, taking the total cross sectional area of the stained artery (Figure 7). The cell nuclei quantification was made only considering the media layer of each artery.

**TABLE 4:** Cell nuclei counts every 100 μm^2^: **(A)** aorta artery, **(B)** carotid and femoral arteries.

(A)									

Arterial segment	ATA (ascending thoracic aorta)			PAA (proximal abdominal artery)			DAA (distal abdominal artery)		
Group	Control	ANP	Cinaciguat	Control	ANP	Cinaciguat	Control	ANP	Cinaciguat
Nucleus	29.56 ± 0.97	29.91 ± 0.71	30.47 ± 0.66	30.63 ± 0.63 (*, +)	24.38 ± 1.48(*)	27.93 ± 0.89(+)	31.38 ± 0.57	30.44 ± 0.39	32.00 ± 0.71

**(B)**									

**Arterial segment**	**Distal carotid artery (DCA)**			**Proximal carotid artery (PCA)**			**Femoral (FA)**		
**Group**	**Control**	**ANP**	**Cinaciguat**	**Control**	**ANP**	**Cinaciguat**	**Control**	**ANP**	**Cinaciguat**

Nucleus	31.82 ± 0.60(*, +)	29.00 ± 1.20(*)	23.75 ± 1.51(+)	30.96 ± 0.48(*)	30.47 ± 0.64	28.79 ± 0.67(*)	46.20 ± 2.88(*)	41.00 ± 1.36	38.43 ± 1.59(*)

ATA, Ascending thoracic aorta; PAA, Proximal abdominal aorta; DAA, Distal abdominal aorta; FA, Femoral artery; PCA, Proximal carotid artery; DCA, Distal carotid artery. Values are expressed as mean ± SEM. Significant differences (p ≤ 0.05, t-student): a matching pair of symbols (* and +) indicate the groups that present differences.

In Tables 4A,B significant differences in cell nuclei content between treated and untreated groups are seen in the aorta (specifically in the PAA zone) and distal carotid (DCA). Both ANP and Cinaciguat groups show increased values relative to the control group. Moreover, the Cinaciguat group presents significant differences in cell nuclei content on the carotid and femoral arteries.

To quantify the content of muscle tissue in the arteries and to determine potential changes of this variable between the studied groups, the ratio of muscle thickness to some geometric parameter of the artery is studied. Specifically, Table 5 shows the percentage proportion between muscle thickness and radial measurement of the analyzed arteries (both external and internal radii). However, there are no changes in the muscle tissue content of the arteries.

**TABLE 5:** Percentage ratio between muscle thickness and external and internal arterial radius equivalent in studied arteries according to the t-student analysis (in parentheses).

	% muscle thickness/external radius			% muscle thickness/internal radius		
Artery	Control	ANP	Cinaciguat	Control	ANP	Cinaciguat
ATA	34.53 ± 5.11	33.89 ± 1.53	35.16 ± 5.48	47.56 ± 7.63	50.81 ± 2.59	53.87 ± 10.45
		(0.91)	(0.93)		(0.70)	(0.64)
PAA	26.34 ± 5.30	20.33 ± 1.19	17.28 ± 1.09	37.63 ± 9.66	26.38 ± 1.80	21.13 ± 1.33
		(0.31)	(0.15)		(0.30)	(0.14)
DAA	16.67 ± 1.02	17.03 ± 2.75	14.64 ± 2.16	20.94 ± 1.68	22.67 ± 4.10	18.20 ± 3.15
		(0.74)	(0.43)		(0.62)	(0.44)
Proximal carotid (PCA)	15.33 ± 2.28	17.79 ± 3.21	16.85 ± 1.32	19.66 ± 3.25	24.19 ± 4.13	21.81 ± 2.32
		(0.56)	(0.62)		(0.42)	(0.64)
Distal carotid (DCA)	22.65 ± 3.40	30.90 ± 1.67	24.52 ± 2.80	31.28 ± 5.77	47.37 ± 3.38	35.54 ± 5.77
		(0.06)	(0.73)		(0.06)	(0.65)
Femoral (FA)	12.42 ± 3.24	21.33 ± 2.11	13.46 ± 2.47	16.55 ± 5.09	32.83 ± 6.58	18.70 ± 3.94
		(0.06)	(0.81)		(0.13)	(0.76)

*ATA, Ascending thoracic aorta; PAA, Proximal abdominal aorta; DAA, Distal abdominal aorta; FA, Femoral artery; PCA, Proximal carotid artery; DCA, Distal carotid artery. Values are expressed as mean ± SEM. In addition, p-value from t-student test was indicated. There are not significant differences (p ≤ 0.05) in any case.*

## Discussion

According to statistical analysis, global physiological pre-stretching in the aorta, carotid, and femoral arteries (Figures 5A,C,E), do not show significant differences between all groups. Making a localized analysis of pre-stretching (Figures 5B,D,F), going from the proximal to the distal regions, it can be stated that the global behavior is replicated in most of the analyzed zones. Figure 5B shows the previously mentioned trend, because in all sectors, except in the last one, the average pre-stretching value is lower in the treated groups compared to the control group. In Figure 5D a similar behavior is seen, except for the proximal sector. In fact, more noticeable differences are found in the distal zone neighborhood, where the decline of the pre-stretching values is statistically significant when the treatments are applied. In the same way, with respect to previously mentioned cases, Figure 5F shows that the ANP and Cinaciguat groups generally decrease the pre-stretching values in the femoral artery (FA), with the exception of the distal zone, compared to the Control group. In relation to circumferential residual stress in aorta artery (Figure 6A), which can be quantified by the ring opening angle results, there is a marked trend to decrease its value when the ANP and Cinaciguat treatments are applied, where this difference is noticeable marked in the PAA zone, with statistically significant differences in both treatments. In this sense, a similar trend is observed in the work of Rivera et al. (2020), who using the same animal model of sheep subjected to chronic hypoxia conditions and treated with melatonin, report a marked difference in the opening angle of the thoracic aorta (an average of 144.0° in the control group) and the abdominal aorta (an average of 50.6° in the control group); however, in the mentioned work, an analysis of the PAA zone is not performed, so there is no information on the effect of melatonin treatment in that intermediate zone. Fung and Liu (1991) show the evolution of the opening angle along the aortic duct in normoxic and hypertensive rats, where for the former case, a diminution of the opening angle is observed going toward the middle zone of the aortic artery (PAA), aspect that is replicated in the opening angle of the groups subjected to ANP and Cinaciguat drug in this study (Figure 6A). In the carotid artery, the same behavior is seen in both the proximal (PCA) and distal (DCA) zones (Figure 6B). The only difference in behavior is seen in the femoral artery (FA) when applying the ANP drug, where the opening angle increases, although this difference is not statistically significant (Figure 6C). Correlation analysis between fiber densities and opening angle measurements reveal some degree of dependency of elastin and collagen fibers over the resulting opening angle of each artery (Figure 8), taking for this analysis the data over all the zones of the aorta artery (ATA, PAA, DAA). These results suggest that the opening angle values at a stress-free configuration are been constrained by the artery microstructural changes. Tian et al. (2011), describes some similar opening angle and stiffness behavior. However, no other biomechanical or histological variables are statistically correlated between them. Therefore, from the pre-stretching and ring opening tests, there is a clear trend to relaxation of the vascular tissues when any of the treatments is applied, which is a desirable outcome to decrease the effects of hypoxia-induced pulmonary hypertension.

On the other hand, in contrast with the mechanical response, histological results do not present morphostructural changes in the thickness of the media and adventitious layers, as well in the vascular lumen with ANP treatment; however, a decrement in collagen deposits of PAA and DAA zones (Table 3C). According to the study by Ooshima et al. (1974), an increment in collagen production of blood vessels in hypertensive rats (particularly in the aorta artery), and a subsequent decrease in this effect through the application of antihypertensive agents, is reported. Therefore, the effect observed in Table 3C, where the ANP drug generates a decrease in collagen content in PAA and DAA zones, is in accordance with the expected effect of this treatment, appeasing the proliferative effects of collagen generated by the condition of chronic hypoxia. Accumulated experimental data shows that a decrease in oxygen tension (hypoxia) is an independent factor regulating the synthesis and release of natriuretic peptides (Arjamaa and Nikinmaa, 2011). However, chronic hypoxia can cause a decrease in intracellular ATP, which causes desensitization of ANP receptors (NPR-A, NPR-B, and NPR-C (Gaur et al., 2018) decreasing receptor activation, beside, another method for removal of ANP is through enzymatic degradation by membrane bound neural endopeptidase (NEP) the activation of this protein decreases the bioavailability of ANP, decreasing its biological action, as demonstrated by models of pulmonary hypertension due to chronic hypoxia (Misono et al., 2011). Analyzing only the Cinaciguat treatment and its effect in the amount of cell nuclei, the PAA and distal carotid (DCA), the proximal carotid (PCA) and femoral arteries (FA) also exhibit a significant drop in cell nuclei density (Table 4). However, except for media thickness in the proximal abdominal aortic artery (PAA) and collagen deposits in femoral artery (FA), no changes were observed in the vascular morphostructure of the different territories analyzed (Tables 3A,D). The purpose of administration of cinaciguat or that of soluble guanylate cyclase activators, is to generate cGMP independent of the redox state of the enzyme, however, the molecule of cinaciguat (BAY-418543) or YC-1 they have almost no inhibitory effect on phosphodiesterase 5 (PDE-5), which generates a decrease in the bioavailability of cGMP at the concentrations needed to stimulate sGC (Horst and Marletta, 2018), this effect explains the differential effect of Cinaciguat in the different vascular territories analyzed. Furthermore, studies have shown the induction of the PDE-5 enzyme associated with hypoxia, which would increase the degradation of the generated cGMP (Nydegger et al., 2019).

Contrasting the results of this study with those obtained in related research, potential effects between cell stiffness and arterial tissue stiffness can be correlated. In this sense Glagov et al. (1987), through isolated cells by Atomic Force Microscopy (AFM), state that the main contribution to vascular wall stiffness is due to cell nuclei stiffness, because the elastin matrix is not enough to generate changes in these properties by itself. The previously mentioned drop of internal deformation and its relationship with a lower cell nuclei density is triggered by the application of ANP and Cinaciguat. From this fact it is possible to infer that internal stress state configuration is lower in the treated groups compared to the control group. This implies that the analyzed treatments induce a relaxation state in arterial tissue, which could mitigate the effects caused by hypoxia-induced pulmonary hypertension. It is also seen that there is no significant difference between the arterial geometry of freshly removed tissues and the studied groups (Table 2). This, added to the fact that the cell nuclei decreases due to the application of drugs allows to indicate that they have an antiproliferative effect, which has been supported by previous studies, by means of drugs used in hypertension pulmonary cases related to hypoxia condition (Glagov et al., 1987). The antiproliferative effect mentioned earlier can impact vascular remodeling, tending to improved vascular function and vasodilator capacity (Glagov et al., 1987).

Histomorphometry results obtained in this study, differ respect to studies carried out in similar arteries for different animal models. Tonar et al. (2015) who carry out their study in the aortic artery in domestic pigs (age 0–230 days), establish that the elastin content is maximum in the descending thoracic aorta (ATA zone), decreasing to a minimum going to DAA zone. These results are inversely correlated with those obtained in this work, where the elastin content is higher in abdominal zone (PAA and DAA). A similar trend is observed for collagen content, where Tonar et al. (2015), report an increase in this microconstituent going from the ascending thoracic (ATA) to the proximal abdominal zone (PAA), which also differs from the trend observed in this work. Regard to vascular SMCs markers in abdominal zone are highest, which can be related with high cell nuclei density in DAA determined in this work. Tomasek et al. (2020) perform a similar study in porcine common carotid artery aged 12–21 weeks, through of an extensive mapping along its length. Comparing main results with the results obtained in this work, in both a decreasing of elastin content from proximal to distal zones can be observed. Respect to collagen content there is not reported a distinguishable tendency in Tomasek et al. (2020), unlike this work, where an increment in the percentage of collagen is observed from proximal to distal zone. Therefore, from information provided by literature, it can be indicated that there is no a marked correlation in the histological results for the different arteries, which can be explained by the use of different animal models and by the condition of chronic hypoxia studied in this work.

Another remarkable issue to consider is the categorization according to the type of artery (muscular or elastic artery taking account its microconstituents. The muscular arteries are mainly characterized by having a higher density of SMCs, which is directly related to the nuclei density reported in Table 4. In this sense, femoral artery (FA) presents a higher density of nucleus respect to the aorta and carotid arteries. This result is supported by Witter et al. (2017), who classifies the femoral artery of pigs as muscular type, although for other animal models the femoral presents an intermediate characteristic between elastic and muscular type (hybrid state). On the other hand, a high content of collagen and elastin characterizes an elastic artery. From Table 3, it is observed that percentage of collagen and elastin is higher in the aorta artery, particularly in its middle and distal area (PAA and DAA), which is in accordance with this type of arteries, as previously mentioned and indicated by Witter et al. (2017). However, a low elastin and collagen content is observed in the ATA, which is not expected due to the elastic nature of the aorta, which could be a consequence of the effect of chronic hypoxia. The carotid artery does not present any particularly high or low levels of collagen, elastin and nucleus, it is categorized in an hybrid artery, as reported by Witter et al. (2017). Further studies need to evaluate age and oxygenation as determinants of the collagen and elastin components to have a definitive understanding of the mechanisms that determine the arterial characteristics in our model.

The studied arteries were obtained from 15 days old newborn sheep (Ovis aries). Although the turnover of extracellular matrix (ECM) is unknown in sheep, studies in humans or rats vary from 12 to 60 days (Martufi and Gasser, 2012). Therefore, we presume that the turnover during the 2 first weeks of life is not an issue in this model. However, it is widely accepted that changes in ECM protein expression often provide insight into the pathological state of cells. For instance, pulmonary arteries from PPHN sheep had increased mechanical stiffness and altered ECM remodeling compared with control normoxic animal (Dodson et al., 2014a). These authors demonstrated that PPHN vessels have a smaller contribution of elastin and a greater role for collagen fiber engagement compared with the control arteries. Furthermore, Dodson et al. (2014b) demonstrated the same pattern in aortas from growth restricted fetuses. In addition, decreased elastin and collagen expression in SMCs is indicative of a phenotypic switch from the contractile state to a synthetic phenotype. This switch from the contractile phenotype to the synthetic phenotype presents a challenge when engineering vessels, as the inadequate elastin and collagen deposition by synthetic SMCs often compromises the mechanical integrity of vessel constructs, characteristics of remodeling associated with chronic hypoxia. Although we do not have a normoxic control in our experiments, there is enough evidence of the vascular remodeling induced in hypoxia, deriving in vascular increased collagen deposition and stiffness during the neonatal stage.

Future research should be aimed at studying the effects of different oxygenation levels and new treatments on the mechanical response of the arterial wall. Moreover, to get more information about the material behavior of the artery, new experimental procedures should be incorporated, making, for example, tensile tests on the tissues to quantify the mechanical response and relate it to histological measurements such as changes in cell nuclei percentage.

## Data Availability Statement

The original contributions presented in the study are included in the article/Supplementary Material, further inquiries can be directed to the corresponding author.

## Ethics Statement

The animal study was reviewed and approved by the Faculty of Medicine Ethics Committee of the Universidad de Chile approved all experimental procedures (Protocol CBA#694 FMUCH).

## Author Contributions

AN, CG-H, EAH, and AJL developed the concept and design of the work. ZC, DP, AG-C, and EAH performed pre-stretching and ring opening tests. AN, ZC, PA, and AU, performed post-processing data from these tests. RVR and FAB performed histological procedures. AN, ZC, and AU determined and analyzed histological results. GE, RVR, AJL, and EAH provided suggestions and editing assistance. All authors made substantial contributions to this work, critically revised the manuscript and approved the final version.

## Conflict of Interest

The authors declare that the research was conducted in the absence of any commercial or financial relationships that could be construed as a potential conflict of interest.

## References

[B1] Abdalla S. S. Will J. A. (1995). Effects of hypoxia, mechanical and chemical endothelium denudation on guinea-pig isolated pulmonary arteries. *Gen. Pharmacol.* 26 113–122. 10.1016/0306-3623(94)00165-J 7713350

[B2] Arjamaa O. Nikinmaa M. (2011). Hypoxia regulates the natriuretic peptide system. *Int. J. Physiol. Pathophysiol. Pharmacol.* 3 191–201. 21941610 PMC3175745

[B3] Astorga C. R. González-Candia A. Candia A. A. Figueroa E. G. Cañas D. Ebensperger G. (2018). Melatonin decreases pulmonary vascular remodeling and oxygen sensitivity in pulmonary hypertensive Newborn Lambs. *Front. Physiol.* 9:185 10.3389/fhys.2018.00185 PMC5845624 29559926

[B4] Brossollet L. J. Vito R. P. (1995). An alternate formulation of blood vessel mechanics and the meaning of the in vivo property. *J. Biomech.* 28 679–687. 10.1016/0021-9290(94)00119-O 7601867

[B5] Cañas D. García-Herrera C. M. Herrera E. A. Celentano D. J. Krause B. J. (2018). Mechanical characterization of arteries affected by fetal growth restriction in guinea pigs (Cavia porcellus). *J. Mech. Behav. Biomed. Mater* 88 92–101. 10.1016/j.jmbbm.2018.08.010 30142566

[B6] Cañas D. Herrera E. A. García-Herrera C. Celentano D. Krause B. J. (2017). Fetal growth restriction induces heterogeneous effects on vascular biomechanical and functional properties in guinea pigs (*Cavia porcellus*). *Front. Physiol.* 8:00144. 10.3389/fphys.2017.00144 28344561 PMC5344887

[B7] Castillo-Galán S. Quezada S. Moraga F. A. Ebensperger G. Herrera E. A. Beñaldo F. (2016). 2-Aminoethyldiphenylborinate modifies the pulmonary circulation in pulmonary hypertensive newborn lambs partially gestated at high altitude. *Am. J. Physiol. Lung Cell. Mol. Physiol.* 311 L788–L799. 10.1152/ajplung.00230.2016 27542806

[B8] Chen Y. F. Feng J. A. Li P. Xing D. Zhang Y. Serra R. (2006). Dominant negative mutation of the TGF-β eceptor blocks hypoxia-induced pulmonary vascular remodeling. *J. Appl. Physiol.* 100 564–571. 10.1152/japplphysiol.00595.2005 16223981

[B9] Chester M. Seedorf G. Tourneux P. Gien J. Tseng N. Grover T. (2011). Cinaciguat, a soluble guanylate cyclase activator, augments cGMP after oxidative stress and causes pulmonary vasodilation in neonatal pulmonary hypertension. *Am. J. Physiol. Lung Cell. Mol. Physiol.* 301:L755. 10.1152/ajplung.00138.2010 21856817 PMC3213988

[B10] Chester M. Tourneux P. Seedorf G. Grover T. R. Gien J. Abman S. H. (2009). Cinaciguat, a soluble guanylate cyclase activator, causes potent and sustained pulmonary vasodilation in the ovine fetus. *Am. J. Physiol. – Lung Cell. Mol. Physiol.* 297:L318. 10.1152/ajplung.00062.2009 19465519 PMC2742799

[B11] Chetan G. Rathisharmila R. Narayanan P. Vishnu Bhat B. (2007). Persistent pulmonary hypertension of the newborn. *Biomedicine* 27 136–142. 10.3390/children4080063 28788074 PMC5575585

[B12] de Gelidi S. Tozzi G. Bucchi A. (2017). The effect of thickness measurement on numerical arterial models. *Mater. Sci. Eng. C* 76 1205–1215. 10.1016/j.msec.2017.02.123 28482487

[B13] Destrade M. Lusetti I. Mangan R. Sigaeva T. (2017). Wrinkles in the opening angle method. *Int. J. Solids Struct.* 122–123 189–195.

[B14] Dodson R. B. Morgan M. R. Galambos C. Hunter K. S. Abman S. H. (2014a). Chronic intrauterine pulmonary hypertension increases main pulmonary artery stiffness and adventitial remodeling in fetal sheep. *Am. J. Physiol. Lung Cell. Mol. Physiol.* 307 L822–L828. 10.1152/ajplung.00256.2014 25326575 PMC4254964

[B15] Dodson R. B. Rozance P. J. Petrash C. C. Hunter K. S. Ferguson V. L. (2014b). Thoracic and abdominal aortas stiffen through unique extracellular matrix changes in intrauterine growth restricted fetal sheep. *Am. J. Physiol. Hear. Circ. Physiol.* 306 H429–437. 10.1152/ajpheart.00472.2013 24322609 PMC3920138

[B16] Fuloria M. Aschner J. L. (2017). Persistent pulmonary hypertension of the newborn. *Semin. Fetal Neonatal Med.* 22 220–226. 10.1016/j.siny.2017.03.004 28342684

[B17] Fung Y. C. Liu S. Q. (1991). Changes of zero-stress state of rat pulmonary arteries in hypoxic hypertension. *J. Appl. Physiol.* 70 2455–2470. 10.1152/jappl.1991.70.6.2455 1885439

[B18] Gao Y. Raj J. U. (2011). Hypoxic pulmonary hypertension of the newborn. *Compr. Physiol.* 1 61–79. 10.1002/cphy.c090015 23737164

[B19] García-Herrera C. M. Bustosa C. A. Celentano D. J. Ortega R. (2016). Mechanical analysis of the ring opening test applied to human ascending aortas. *Comput. Methods Biomech. Biomed. Engin.* 19 1738–1748. 10.1080/10255842.2016.1183125 27178265

[B20] García-Herrera C. M. Celentano D. J. Cruchaga M. A. Rojo F. J. Atienza J. M. Guinea G. V. (2012). Mechanical characterisation of the human thoracic descending aorta: Experiments and modelling. *Comput. Methods Biomech. Biomed. Engin.* 15 185–193. 10.1080/10255842.2010.520704 21480018

[B21] Gaur P. Saini S. Vats P. Kumar B. (2018). Regulation, signalling and functions of hormonal peptides in pulmonary vascular remodelling during hypoxia. *Endocrine* 59 466–480. 10.1007/s12020-018-1529-0 29383676

[B22] Glagov S. Weisenberg E. Zarins C. K. Stankunavicius R. Kolettis G. J. (1987). Compensatory Enlargement of Human Atherosclerotic Coronary Arteries. *N. Engl. J. Med.* 316 1371–1375. 10.1056/NEJM198705283162204 3574413

[B23] Golob M. J. Tabima D. M. Wolf G. D. Johnston J. L. Forouzan O. Mulchrone A. M. (2017). Pulmonary arterial strain- and remodeling-induced stiffening are differentiated in a chronic model of pulmonary hypertension. *J. Biomech.* 55 92–98. 10.1016/j.jbiomech.2017.02.003 28262286 PMC5535793

[B24] Gonzaléz-Candia A. Candia A. A. Ebensperger G. Reyes R. V. Llanos A. J. Herrera E. A. (2020). The newborn sheep translational model for pulmonary arterial hypertension of the neonate at high altitude. *J. Dev. Orig. Health and Dis.* 11 452-463. 10.1017/S2040174420000616 32705972

[B25] Herrera E. A. Camm E. J. Cross C. M. Mullender J. L. Wooding F. B. P. Giussani D. A. (2011). Morphological and functional alterations in the aorta of the chronically hypoxic fetal rat. *J. Vasc. Res.* 49 50–58. 10.1159/000330666 21985843

[B26] Herrera E. A. Farías J. G. Ebensperger G. Reyes R. V. Llanos A. J. Castillo R. L. (2015). Pharmacological approaches in either intermittent or permanent hypoxia: A tale of two exposures. *Pharmacol. Res.* 101 94–101. 10.1016/j.phrs.2015.07.011 26215469

[B27] Herrera E. A. Riquelme R. A. Ebensperger G. Reyes R. V. Ulloa C. E. Cabello G. (2010). Long-term exposure to high-altitude chronic hypoxia during gestation induces neonatal pulmonary hypertension at sea level. *Am. J. Physiol. Regul. Integr. Comp. Physiol.* 299:R1676. 10.1152/ajpregu.00123.2010 20881096 PMC3007194

[B28] Horný L. Adámek T. Kulvajtová M. (2017). A comparison of age-related changes in axial prestretch in human carotid arteries and in human abdominal aorta. *Biomech. Model. Mechanobiol.* 16 375–383. 10.1007/s10237-016-0797-y 27189696

[B29] Horst B. G. Marletta M. A. (2018). Physiological activation and deactivation of soluble guanylate cyclase. *Nitric Oxide Biol. Chem.* 77 65–74. 10.1016/j.niox.2018.04.011 29704567 PMC6919197

[B30] Huicho L. Niermeyer S. (2006). Cardiopulmonary pathology among children resident at high altitude in Tintaya, Peru: A cross-sectional study. *High Alt. Med. Biol.* 7 168–179. 10.1089/ham.2006.7.168 16764529

[B31] Julian C. G. (2011). High Altitude During Pregnancy. *Clin. Chest Med.* 32 21–31. 10.1016/j.ccm.2010.10.008 21277446

[B32] Karimi A. Navidbakhsh M. Shojaei A. Faghihi S. (2013). Measurement of the uniaxial mechanical properties of healthy and atherosclerotic human coronary arteries. *Mater. Sci. Eng. C* 33 2550–2554. 10.1016/j.msec.2013.02.016 23623067

[B33] Ke J. Wang L. Xiao D. (2017). “Cardiovascular Adaptation to High-Altitude Hypoxia,” in *Hypoxia and Human Diseases*, eds Jun K. Lei W. Daliao X. (London: InTechopen).

[B34] Keyes L. E. Armaza J. F. Niermeyer S. Vargas E. Young D. A. Moore L. G. (2003). Intrauterine growth restriction, preeclampsia, and intrauterine mortality at high altitude in Bolivia. *Pediatr. Res.* 54 20–25. 10.1203/01.PDR.0000069846.64389.DC 12700368

[B35] Kochová P. Kuncová J. Svíglerová J. Cimrman R. Miklíková M. Liška V. (2012). The contribution of vascular smooth muscle, elastin and collagen on the passive mechanics of porcine carotid arteries. *Physiol. Meas.* 33 1335–1351. 22813960 10.1088/0967-3334/33/8/1335

[B36] Labrosse M. R. Beller C. J. Mesana T. Veinot J. P. (2009). Mechanical behavior of human aortas: Experiments, material constants and 3-D finite element modeling including residual stress. *J. Biomech.* 42 996–1004. 10.1016/j.jbiomech.2009.02.009 19345356

[B37] Li Z. Huang W. Jiang Z. L. Gregersen H. Fung Y. C. (2004). Tissue remodeling of rat pulmonary arteries in recovery from hypoxic hypertension. *Proc. Natl. Acad. Sci. U. S. A.* 101 11488–11493. 10.1073/pnas.0404084101 15277667 PMC509227

[B38] Liu S. Q. Fung Y. C. (1988). Zero-stress states of arteries. *J. Biomech. Eng.* 110 82–84. 10.1115/1.3108410 3347028

[B39] Martufi G. Gasser T. C. (2012). Turnover of fibrillar collagen in soft biological tissue with application to the expansion of abdominal aortic aneurysms. *J. R. Soc. Interface* 9 3366–3377. 10.1098/rsif.2012.0416 22896562 PMC3481568

[B40] Misono K. S. Philo J. S. Arakawa T. Ogata C. M. Qiu Y. Ogawa H. (2011). Structure, signaling mechanism and regulation of the natriuretic peptide receptor guanylate cyclase. *FEBS J.* 278 1818–1829. 10.1111/j.1742-4658.2011.08083.x 21375693 PMC3097287

[B41] Morrison J. L. Berry M. J. Botting K. J. Darby J. R. T. Frasch M. G. Gatford K. L. (2018). Improving pregnancy outcomes in humans through studies in sheep. *Am. J. Physiol. Regul. Integr. Comp. Physiol.* 315 R1123–R1153. 10.1152/ajpregu.00391.2017 30325659

[B42] Nydegger C. Corno A. F. von Segesser L. K. Beghetti M. Samaja M. Milano G. (2019). Effects of PDE-5 Inhibition on the Cardiopulmonary System After 2 or 4 Weeks of Chronic Hypoxia. *Cardiovasc. Drugs Ther.* 33 407–414. 10.1007/s10557-019-06887-9 31264002 PMC6689028

[B43] Ooshima A. Fuller G. C. Cardinale G. J. Spector S. Udenfriend S. (1974). Increased collagen synthesis in blood vessels of hypertensive rats and its reversal by antihypertensive agents. *Proc. Natl. Acad. Sci. U. S. A.* 71 3019–3023. 10.1073/pnas.71.8.3019 4370097 PMC388611

[B44] Palmer S. K. Moore L. G. Young D. A. Cregger B. Berman J. C. Zamudio S. (1999). Altered blood pressure course during normal pregnancy and increased preeclampsia at high altitude (3100 meters) in Colorado. *Am. J. Obstet. Gynecol.* 180 1161–1168. 10.1016/S0002-9378(99)70611-3 10329872

[B45] Pandey K. N. (2005). Biology of natriuretic peptides and their receptors. *Peptides* 26 901–932. 10.1016/j.peptides.2004.09.024 15911062

[B46] Penaloza D. (2012). Effects of High-Altitude Exposure on the Pulmonary Circulation. *Rev. Española Cardiol.* 65 1075–1078. 10.1016/j.rec.2012.06.017 23068616

[B47] Penaloza D. Arias-Stella J. (2007). The heart and pulmonary circulation at high altitudes: Healthy highlanders and chronic mountain sickness. *Circulation* 115 1132–1146. 10.1161/CIRCULATIONAHA.106.624544 17339571

[B48] Pichamuthu J. E. Phillippi J. A. Cleary D. A. Chew D. W. Hempel J. Vorp D. A. (2013). Differential tensile strength and collagen composition in ascending aortic aneurysms by aortic valve phenotype. *Ann. Thorac. Surg.* 96 2147–2154. 10.1016/j.athoracsur.2013.07.001 24021768 PMC4016718

[B49] Rachev A. Manoach E. Berry J. Moore J. E. (2000). A model of stress-induced geometrical remodeling of vessel segments adjacent to stents and artery/graft anastomoses. *J. Theor. Biol.* 206 429–443. 10988028 10.1006/jtbi.2000.2143

[B50] Rivera E. García-Herrera C. González-Candia A. Celentano D. J. Herrera E. A. (2020). Effects of melatonin on the passive mechanical response of arteries in chronic hypoxic newborn lambs. *J. Mech. Behav. Biomed. Mater.* 112:2020. 10.1016/j.jmbbm.2020.104013 32846285

[B51] Rol N. Timmer E. M. Faes T. J. Vonk Noordegraaf A. Grünberg K. Bogaard H. J. (2017). Vascular narrowing in pulmonary arterial hypertension is heterogeneous: rethinking resistance. *Physiol. Rep.* 5:e13159. 10.14814/phy2.13159 28320897 PMC5371554

[B52] Rouwet E. V. Tintu A. N. Schellings M. W. van Bilsen M. Lutgens E. Hofstra L. (2002). Hypoxia Induces Aortic Hypertrophic Growth, Left Ventricular Dysfunction, and Sympathetic Hyperinnervation of Peripheral Arteries in the Chick Embryo. *Circulation* 105 2791–2796. 10.1161/01.CIR.0000017497.47084.06 12057996

[B53] Salinas C. E. Blanco C. E. Villena M. Camm E. J. Tuckett J. D. Weerakkody R. A. (2010). Cardiac and vascular disease prior to hatching in chick embryos incubated at high altitude. *J. Dev. Orig. Health Dis.* 1 60–66. 10.1017/S2040174409990043 25142932

[B54] Sehgel N. L. Vatner S. F. Meininger G. A. (2015). Smooth muscle cell stiffness syndrome’-Revisiting the structural basis of arterial stiffness. *Front. Physiol.* 6:335. 10.3389/fphys.2015.00335 26635621 PMC4649054

[B55] Sharma V. Berkelhamer S. Lakshminrusimha S. (2015). Persistent pulmonary hypertension of the newborn. *Matern. Heal. Neonatol. Perinatol.* 1:14. 10.1186/s40748-015-0015-4 27057331 PMC4823682

[B56] Skilton M. R. Evans N. Griffiths K. A. Harmer J. A. Celermajer D. S. (2005). Aortic wall thickness in newborns with intrauterine growth restriction. *Lancet* 365 1484–1486. 10.1016/S0140-6736(05)66419-7 15850633

[B57] Steinhorn R. H. (2010). Neonatal pulmonary hypertension. *Pediatr. Crit. Care Med.* 11 S79–S84. 10.1097/PCC.0b013e3181c76cdc 20216169 PMC2843001

[B58] Su J. Logan C. C. Hughes A. D. Parker K. H. Dhutia N. M. Danielsen C. C. (2018). Impact of chronic hypoxia on proximal pulmonary artery wave propagation and mechanical properties in rats. *Am. J. Physiol. Hear. Circ. Physiol.* 314 H1264–H1278. 10.1152/ajpheart.00695.2017 29547024 PMC6032080

[B59] Tan A. C. I. T. L. Russel F. G. M. Thien T. Benraad T. J. (1993). Atrial Natriuretic Peptide: An Overview of Clinical Pharmacology and Pharmacokinetics. *Clin. Pharmacokinetics* 24 28–45. 10.2165/00003088-199324010-00003 8448971

[B60] The Cleveland Clinic (2020). *Hipertensión Pulmonar: Causas, Síntomas, Diagnóstico, Tratamiento.* http://www.clevelandclinic.org/health/sHIC/html/s6530.asp (accessed April 03, 2020) Cleveland: The Cleveland Clinic.

[B61] Thompson J. A. Richardson B. S. Gagnon R. Regnault T. R. H. (2011). Chronic intrauterine hypoxia interferes with aortic development in the late gestation ovine fetus. *J. Physiol.* 589 3319–3332. 10.1113/jphysiol.2011.210625 21540340 PMC3145942

[B62] Tian L. Lammers S. R. Kao P. H. Reusser M. Stenmark K. R. Hunter K. S. (2011). Linked opening angle and histological and mechanical aspects of the proximal pulmonary arteries of healthy and pulmonary hypertensive rats and calves. *Am. J. Physiol. Hear. Circ. Physiol* 301 H1810–818. 10.1152/ajpheart.00025.2011 21856906 PMC3213979

[B63] Tomasek P. Tonar Z. Grajciarová M. Kural T. Turek D. Horáková J. (2020). Histological mapping of porcine carotid arteries – An animal model for the assessment of artificial conduits suitable for coronary bypass grafting in humans. *Ann. Anat.* 228 151434. 10.1016/j.aanat.2019.151434 31704146

[B64] Tonar Z. Kubíková T. Prior C. Demjén E. Liška V. Králíèková M. (2015). Segmental and age differences in the elastin network, collagen, and smooth muscle phenotype in the tunica media of the porcine aorta. *Ann. Anat.* 201 79–90. 10.1016/j.aanat.2015.05.005 26232584

[B65] Tschanz S. Schneider J. P. Knudsen L. (2014). Design-based stereology: Planning, volumetry and sampling are crucial steps for a successful study. *Ann. Anat.* 196 3–11. 10.1016/j.aanat.2013.04.011 23769130

[B66] Virués-Ortega J. Garrido E. Javierre C. Kloezeman K. C. (2006). Human behaviour and development under high-altitude conditions. *Dev. Sci.* 9 400–410. 10.1111/j.1467-7687.2006.00505.x 16764613

[B67] Vonavková T. Horný L. (2020). Effect of axial prestretch and adipose tissue on the inflation-extension behavior of the human abdominal aorta. *Comput. Methods Biomech. Biomed. Engin.* 23 81–91. 31814443 10.1080/10255842.2019.1699544

[B68] Wang Z. Lakes R. S. Golob M. Eickhoff J. C. Chesler N. C. (2013). Changes in large pulmonary arterial viscoelasticity in chronic pulmonary hypertension. *PLoS One* 8:2013. 10.1371/journal.pone.0078569 24223157 PMC3819365

[B69] Witter K. Tonar Z. Schöpper H. (2017). How many Layers has the Adventitia? – Structure of the Arterial Tunica Externa Revisited. *J. Vet. Med. Ser. C Anat. Histol. Embryol.* 46 110–120. 10.1111/ahe.12239 27282337

